# Application of the Ridden Horse Pain Ethogram to 150 Horses with Musculoskeletal Pain before and after Diagnostic Anaesthesia

**DOI:** 10.3390/ani13121940

**Published:** 2023-06-09

**Authors:** Sue Dyson, Danica Pollard

**Affiliations:** 1 The Cottage, Church Road, Market Weston, Diss IP22 2NX, UK; 2 The Rodhams, Rodham Road, Christchurch, Wisbech PE14 9NU, UK; drdee.pollard@gmail.com

**Keywords:** lameness, ridden horse behaviour, foot pain, nerve blocks, saddle fit, lumbosacroiliac joint region pain, proximal suspensory desmopathy, thoracolumbosacral range of motion, trot, canter

## Abstract

**Simple Summary:**

Riders are often frustrated because when they recognise a decline in their horse’s performance, they seek professional advice and are informed that their horse is not lame based on an examination in hand ± on the lunge. The Ridden Horse Pain Ethogram (RHpE) was developed to facilitate the recognition of musculoskeletal pain in horses. An RHpE score of ≥8/24 reflects the likely presence of musculoskeletal pain. The aim of this study was to document observations made during ridden exercise of 150 horses with a history of poor performance, comparing RHpE scores before and after nerve blocks ± change of saddle. The most frequent lameness grade when ridden was 2/8 (range: 0–4); 35% of the horses had no overt lameness but lacked hindlimb impulsion. The most frequent RHpE score was 9/24 (range: 2–15/24), which declined to 2/24 (range: 0–12) after the interventions and was associated with improved gait quality and rideability. Despite recent professional saddle fit, an ill-fitting saddle contributed to poor performance in 37% of the horses. This study highlights the importance of ridden exercise in the investigation of poor performance/ low-grade lameness and the value of the RHpE to verify the presence of musculoskeletal pain. Nerve blocks are vital to determine the source(s) of pain that compromise performance.

**Abstract:**

The Ridden Horse Pain Ethogram (RHpE) was developed to facilitate the recognition of musculoskeletal pain. The aim of this study was to document changes in RHpE scores before and after diagnostic anaesthesia was performed to alleviate pain ± when the saddle was changed. One hundred and fifty horses underwent ridden exercise as part of an investigation of poor performance. The RHpE was applied before and after the interventions. Fifty-two (34.7%) horses exhibited a bilaterally symmetrical short step length and/or restricted hindlimb impulsion and engagement. Fifty-three (35.3%) horses had episodic lameness; only forty-five (30.0%) horses were continuously lame. The median maximum lameness grade when ridden was 2/8 (interquartile range [IQR]: 0–3; range: 0–4). Fifty-six (37.3%) horses had an ill-fitting saddle, which was considered likely to influence performance. The median RHpE scores after the interventions (2/24 [IQR: 1–3, range: 0–12]) were significantly lower than before the interventions (9/24 [IQR: 8–11, range: 2–15]) (Wilcoxon signed-rank z = 10.6, *p* < 0.001). There was no correlation between the RHpE score and maximum lameness grade before diagnostic anaesthesia (Spearman’s rho = 0.09, *p* = 0.262). It was concluded that the absence of overt lameness does not preclude primary musculoskeletal pain. Gait quality and performance can be improved by diagnostic anaesthesia, with substantial reductions in RHpE scores.

## 1. Introduction

There is increasing awareness that horses may show gait abnormalities that compromise performance when ridden that are not apparent when assessed moving in hand [1,2,3,4,5]. This highlights the importance of including ridden exercise when investigating either lameness or poor performance. A low-grade lameness observed in hand or during lungeing may not reflect the source(s) of pain that compromise(s) the horse’s ridden performance. The increasing use of objective gait analysis has prompted discussion about what might constitute the degree of asymmetry that reflects pain-induced gait changes, rather than an inherent asymmetrical movement pattern [6,7,8,9,10,11]. The asymmetry of movement, sometimes referred to as laterality, has been documented not only in adult horses but also in foals [12].

The Ridden Horse Pain Ethogram (RHpE) was developed to facilitate the recognition of pain in ridden horses and comprises 24 behaviours, the majority of which are at least 10 times more likely to be seen in a horse with musculoskeletal pain than in a nonlame horse [13,14,15]. An RHpE score of ≥8/24 reflects the likely presence of musculoskeletal pain. The reduction in RHpE scores after abolishing the pain that is causing lameness or other abnormalities of movement via diagnostic anaesthesia provides evidence of a causal relationship between these behaviours and musculoskeletal pain [16,17]. However, the number of horses (*n* = 31) assessed using the RHpE before and after diagnostic anaesthesia and documented in the literature is small. The association between RHpE scores and an ill-fitting saddle, specifically tight tree points of the saddle, or a rider sitting on the caudal third of the saddle has also been demonstrated [4].

The purpose of this study was to describe the gait and behavioural changes in horses undergoing routine investigations of lameness or poor performance before and after diagnostic anaesthesia, and a change in saddle when indicated, in a larger cohort than has previously been documented.

The aims of the study were to: 1. document the RHpE scores before and after diagnostic anaesthesia ± a change in saddle; 2. determine the frequency of occurrence of each of the 24 behaviours of the RHpE; 3. determine which behaviours of the RHpE were most likely to resolve after the removal of pain; and 4. determine the frequency of occurrence of ill-fitting saddles compromising performance.

It was hypothesised that: 1. the RHpE scores would be lower after diagnostic anaesthesia ± a change in the saddle, and 2. there would be no association between lameness grades and RHpE scores.

## 2. Materials and Methods

This was a prospective interventional study involving 150 horses undergoing routine clinical investigations at a variety of geographical locations in the United Kingdom. The horses included in the study were investigated consecutively for lameness or poor performance and were evaluated when ridden before and after diagnostic anaesthesia ± a change of saddle. The owners gave informed consent for the use of any acquired data for research and education. The study was approved by the Clinical Ethical Review Committee of the Animal Health Trust (AHT29-2015). A detailed history for each horse was acquired, and it was consistently apparent that the rider experienced specific problems with the horse’s ridden performance, which necessitated an appraisal of the horse’s performance during ridden exercise. The age, breed, sex and work discipline (dressage, showjumping, eventing, general-purpose activities [which included horses used for unaffiliated competition] and other [endurance, reining]) of all the horses were documented.

### 2.1. Static Examination

All the horses underwent a comprehensive static examination. This included an assessment of posture and conformation; muscle development, muscle symmetry, muscle tone and muscle pain; range of motion of the cervical and thoracolumbosacral regions; reaction to firm pressure over the cervical articular process joints and the thoracolumbar spinous processes and light pressure in the girth region; reaction to pressure applied over the tubera sacrale; inspection and palpation of the limbs in weight-bearing and non-weight-bearing positions; and evaluation of foot conformation, trimming and shoeing [18].

### 2.2. Dynamic Examination

The dynamic assessment included an evaluation in hand at walk and trot, the horse moving in small circles while walking to the left and to the right, and the horse stepping backwards for at least 10 steps. Lameness and other abnormalities, for example, a short-stepping forelimb gait [19] or the tail held to the left [20], were noted. Distal limb flexion tests were performed on the forelimbs and proximal limb flexion tests were performed on the hindlimbs, each for one minute. Other manipulative tests were performed on selected horses based on the preliminary static clinical assessment.

All horses were lunged in trot and canter in a circle of approximately 12–15 m diameter on a soft surface and at trot on a firm surface, which incorporated a gradient. Lameness and other gait abnormalities were recorded, for example inwards trunk lean; head and neck turned to the outside; inside hindlimb crossed under the trunk during protraction; reduced range of motion of the thoracolumbosacral region [19].

The mouth was inspected for evidence of trauma to the bars or commissures of the mouth or buccal mucosal ulceration [21]. Horses with major lesions that were considered likely to alter ridden performance were not included in the study. All the horses were observed being tacked up and mounted [22,23]. The saddle fit was assessed both without and with a rider [24,25]. The assessor (SJD) had previously undergone training with a Society of Master Saddlers Qualified Saddle Fitter. Major causes of an ill-fitting saddle were recorded (for example, tight tree points, gullet too wide and pommel too low). The bridle and bit fit were assessed [26] and the height of the bit was adjusted if it was too low and likely to come into contact with the canine teeth (104, 204, 304, 404). It was noted if the canons of the bit were too wide and whether the sclera of the left or right eye were visible at rest, which are factors that influence the application of two behaviours of the RHpE [14].

All the horses were evaluated ridden around the periphery of a 20 m × 60 m arena with an all-weather, well-maintained surface. The horses were assessed while walking, trotting and cantering. Circles of 20 m and 10 m diameter (as circular figures of eight) were performed in rising trot. Comparisons were made between sitting on the left (left fore and right hind) or the right (right fore and left hind) diagonals, and between rising and sitting trot. When clinically indicated horses were assessed when ridden to a contact (normal rein tension) and on a long rein (reduced rein tension to encourage the horse to lower its head and neck position and stretch forwards). Dressage and event horses performed all the movements that they were trained to perform (for example, shoulder in and half pass). The horses trained to perform flying changes also performed changes between the right lead and left lead canters. The assessor (SJD) was an equine veterinarian with >40 years of experience with lameness and poor performance investigation, a codeveloper of the RHpE, a rider who has competed at upper national levels in eventing and showjumping and an instructor at the British Horse Society. The assessor stood in one of two corners of the arena, on the left and right reins respectively, so that each horse was evaluated from behind, the side and in front on both reins. If a rider described abnormalities in a horse’s jumping performance, the horse was also assessed jumping. The duration of ridden exercise after warm-up usually varied between 10 and 15 min.

### 2.3. Grading Lameness and Other Observations

Lameness was graded independently under each circumstance in which a horse was examined, using a 0–8 scale (0 = nonlame, 2 = mild, 4 = moderate, 6 = severe (although the limb is loaded) and 8 = nonweightbearing; 1 = very mild (just perceptible), 3 = mild–moderate, 5 = moderately severe and 7 = partial weightbearing are the intermediate grades [27]. Each gait was also described; for example, a lack of hindlimb impulsion and engagement in trot, close temporal and spatial placement of the hindlimbs in canter and a lack of a suspension phase. For the purposes of statistical analysis, the maximum lameness grade was that assigned during ridden exercise when the RHpE was applied before diagnostic anaesthesia. The presence or absence of saddle slip [28], side-to-side oscillation of the saddle or dorsoventral saddle movement (bouncing) [25] were recorded. The thoracic region was reinspected after ridden exercise to determine the distribution of sweating under the saddle, the presence of ruffled hair and the development of any soft tissue swellings.

The presence of teeth grinding or an abnormal respiratory noise during exercise was recorded. In addition, a delayed recovery of the resting respiratory rate after exercise relative to the work intensity and duration, environmental temperature, humidity, body condition score and horse fitness; sweating disproportionately to the work intensity and duration, environmental temperature and humidity, body condition score and horse fitness; and an abnormal posture after exercise (for example, hindlimbs camped out) were documented.

Most of the horses were initially assessed when ridden by their usual rider unless the rider was too apprehensive to ride because of a horse’s previous behaviour or the rider was not present. Approximately 55% of the horses were also ridden by one of several trained, skilled technicians who were used to riding horses before and after diagnostic anaesthesia and describing their observations.

### 2.4. Application of the Ridden Horse Pain Ethogram

The RHpE (Table 1) was applied before diagnostic anaesthesia and after diagnostic anaesthesia in addition to a change in the saddle if the saddle was considered likely to be compromising performance. The order in which the diagnostic anaesthetic techniques were performed and the time at which the saddle was changed to an intervention saddle (usually an early prototype flexible saddle, Smart Saddles, Unit 1, High Grounds Road, Rhodesia, Worksop, S80 3AT, UK) was dependent on each horse’s clinical presentation (Table 2). Diagnostic anaesthesia was performed in a systematic way as previously described [29]. The work pattern and duration of the work performed before and after diagnostic anaesthesia were similar for the individual horses.

The RHpE scores were determined from real-time evaluations of the horses; a separate checklist was completed for each horse before any intervention and after the final interventions. Video recordings were available for approximately half the horses, and real-time observations were verified via a retrospective comparison with the video recordings. For the purposes of the RHpE scores, each horse was assessed when ridden by the same rider before and after diagnostic anaesthesia ± change of the saddle.

The aim with each horse was to resolve lameness and to improve both the quality of the gaits and the rideability of the horse (their responsiveness to the rider’s cues). At each stage of the investigation, the rider was asked to provide feedback on the changes that they felt (for example, more symmetrical rein tension, increased range of motion of the thoracolumbar region, increased fluency of movement and increased responsiveness to cues). In the majority of horses, lameness was abolished, but in a minority, there was persistent lameness or obvious residual abnormalities in their gait (for example a bilaterally short stepping forelimb gait) or deteriorations in forelimb lameness in horses with cervical radiculopathy.

The final diagnoses were achieved by radiography, ultrasonography and magnetic resonance imaging, as indicated by the responses to diagnostic anaesthesia and other clinical features.

### 2.5. Data Analysis

Data recording, cleaning and some graphical recreations were carried out in Microsoft Excel (Version 2010; Microsoft Corporation, Redmond, Washington, DC, USA). STATA (IC version 13; StataCorp. LLC. 2017. Stata Statistical Software: Release 15. College Station, TX, USA) commercial software was subsequently used for all descriptive and inferential statistical analyses. Evidence of statistical significance was accepted when *p* < 0.05.

The horses’ age and RHpE scores before and after diagnostic anaesthesia were not normally distributed (Shapiro–Wilk test *p*-value < 0.05), and alongside the maximum lameness grade, they were described by using medians with the interquartile range (IQR) and range. All the remaining categorical variables were described as proportions and are expressed as percentages.

The Wilcoxon signed-rank test was used to determine if there was a statistically significant difference in the median RHpE scores before and after diagnostic anaesthesia (with/without saddle change). The null hypothesis tested was that median RHpE scores before the interventions would equal the RHpE scores after interventions.

The Spearman rank correlation coefficient was used to investigate whether it was possible to detect a significant correlation between the RHpE score and the maximum lameness grade before diagnostic anaesthesia.

The Mann–Whitney *U* test was used to identify whether there was an association between the RHpE score and the presence of an ill-fitting saddle before diagnostic anaesthesia.

## 3. Results

### 3.1. Descriptive Statistics

The population comprised 150 horses with a median age of 8 years (IQR: 6–11; range: 3–19). The breeds included Warmblood (*n* = 67; 44.7%), Irish Sports Horses (*n* = 26; 17.3%), Warmblood × Thoroughbred (*n* = 20; 13.3%), Other (*n* = 12; 12.0%), Pony (*n* = 11; 7.3%) and Thoroughbred (*n* = 8; 5.3%). There were 98 (65.3%) geldings, 7 (4.7%) stallions and 45 (30.0%) mares. The work disciplines comprised general-purpose (including unaffiliated competition) (*n* = 43; 28.7%), dressage (*n* = 41; 27.3%), eventing (*n* = 36; 24.0%), showjumping (*n* = 28; 18.7%) and other (*n* = 2; 1.3%). The usual riders ranged in ability from competing at a riding-club level to international competitions.

Epaxial muscle hypertension ± pain was detected in 40 (26.7%) horses. Fifty-two (34.7%) horses were nonlame in a conventional sense, with no detectable asymmetry, but they exhibited a bilaterally symmetrical short step length and/or restricted hindlimb impulsion and engagement. Fifty-three (35.3%) horses had episodic lameness and only forty-five (30.0%) horses were continuously lame. Lameness in only one limb was observed in only three (2%) horses, all with forelimb lameness which was only detectable during ridden exercise. 

Left hindlimb lameness predominated in 42 (28.0%) horses and right hindlimb lameness in 33 (22.0%) horses (including one horse which was lame in hand but would not trot more than three steps when ridden). Left forelimb lameness predominated in twelve (8.0%) horses (including one horse that was only lame transiently on the right rein when ridden), and right forelimb lameness predominated in ten (6.7%) horses (including four horses that were only lame when ridden in 10 m diameter circles and one horse with concurrent right hindlimb lameness [not included in the above]). The median maximum lameness grade when ridden was 2/8 (IQR: 0–3; range: 0–4). The three horses with unilateral forelimb lameness had grades of 3, 4 and 4/8. Most horses (146/149, 98.0%) had an abnormal canter (one horse refused to canter). The lameness groups are summarised in Table 3.

Fifty-six (37.3%) horses had an ill-fitting saddle that was considered likely to influence their performance, which justified a change in the saddle as part of the diagnostic intervention, and all of the horses showed improvements in gait ± behaviour after changing the saddle. Sixty-seven (44.7%) horses were ridden by their usual rider for diagnostic anaesthesia and 83 (55.3%) horses were ridden by a trained technician.

### 3.2. Ridden Horse Pain Ethogram Scores

**Figure 1:**
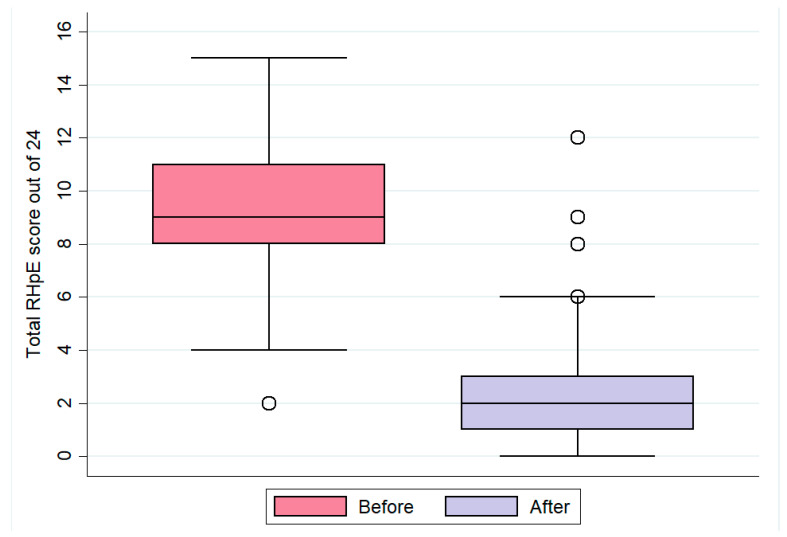
The total Ridden Horse Pain Ethogram (RHpE) scores before and after diagnostic anaesthesia ± saddle change for 150 horses. Boxes represent medians and interquartile ranges; whiskers represent the range, and individual points represent outliers. The median RHpE scores were significantly higher before compared with after diagnostic anaesthesia (Wilcoxon signed-rank z = 10.6, *p* < 0.001).

The median RHpE score for all horses before interventions was 9/24 (IQR: 8–11, range: 2–15). The three horses with unilateral forelimb lameness had scores of 6, 8 and 6/24. After the interventions, the median RHpE score was 2/24 (IQR: 1–3, range: 0–12) (Figure 1). The Wilcoxon signed-rank test revealed that there was a significant difference in the median RHpE scores before and after the interventions (z = 10.6, *p* < 0.001). There were 147 comparisons in which the RHpE score before was greater than the RHpE score after diagnostic anaesthesia ± saddle change, and there was one comparison in which the RHpE score before was lower than the score after and two comparisons in which the two were equal (see also Table 4). These results indicate that diagnostic anaesthesia ± saddle change had a significant effect on lowering the median RHpE scores.

**Figure 2:**
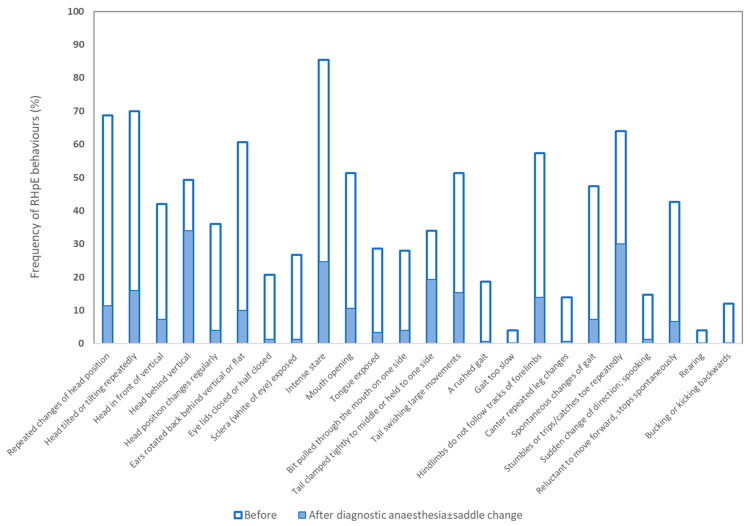
The frequency of each of the 24 behaviours of the Ridden Horse Pain Ethogram (RHpE) in 150 horses before and after diagnostic anaesthesia ± saddle change.

The frequency of occurrence of each behaviour of the RHpE before and after the interventions is illustrated in Figure 2. There were substantial reductions in the frequency of occurrence of each behaviour after diagnostic anaesthesia ± saddle change, with the greatest reductions (difference in % before and after) for intense stare (60.7%), repeated head movement up and down (57.3%), repeated head tilt (54.0%), ears back ≥5 s (50.7%), hindlimbs not following the tracks of the forelimbs (43.3%), mouth opening ≥10 s (40.7%) and spontaneous changes in gait (40.0%) (Table S1). Rearing, bucking and a slow gait were abolished.

There was no significant correlation between the RHpE score and maximum lameness grade before diagnostic anaesthesia (Spearman’s rho = 0.09, *p* = 0.262). The Mann–Whitney *U* test did not identify a significant relationship between the RHpE score and the presence of an ill-fitting saddle before diagnostic anaesthesia (z = −0.91, *p* = 0.365).

### 3.3. Residual Gait Abnormalities

Lameness was abolished and the quality of the gaits was improved in the majority of horses. Six horses had residual gait abnormalities reflecting hindlimb ataxia and weakness. One horse developed moderate hindlimb stumbling after diagnostic anaesthesia of the hindlimbs. Twenty-three horses had residual pain; for example, persistent forelimb lameness attributed to cervical radiculopathy or a bilaterally shortened forelimb step length, which was worse in a canter than in a trot, in association with the severe osteoarthritis of caudal cervical/cranial thoracic articular process joints and neck pain and/or a reduced range of motion (‘stiffness’) (Table 4). One horse had severe psoas major pain, which was determined by rectal palpation. A horse with a severely degenerate lumbosacral intervertebral disk was improved after infiltration of mepivacaine around the sacroiliac joints, but mild discomfort persisted (Table 4, horse 37). Five horses had a suspected component of lumbosacroiliac joint region pain (Table 4, horses 116, 126, 136, 137, 142), two of which developed subtle transient ataxia after infiltration of mepivacaine around the sacroiliac joints and were not reassessed when ridden. The sacroiliac joints were not blocked in the other three horses because the owners did not want to proceed further. Overall, the residual abnormalities of the canter persisted, albeit they were improved compared with the baseline, in 15 (10%) horses.

Two horses in which cervical radiculopathy was the only problem (Table 4, horses 76 and 77) and four horses with suspected cervical radiculopathy and concurrent hindlimb lameness (Table 4, horses 39, 87, 134, 145) had an increase of one in their RHpE score (Table 4, horse 87), no change in their RHpE scores (Table 4, horses 77, 134) or a reduction in their RHpE score by one (horse 76), four (horse 145) or eight (horse 39). The remaining 17 horses with residual pain had a reduction in their RHpE scores ranging from 1 to 10 (median 6) after diagnostic anaesthesia resulted in gait improvement.

**Table 4:** Ridden Horse Pain Ethogram (RHpE) scores before and after diagnostic anaesthesia ± saddle change in 23 horses in which persistent pain-related gait abnormalities were observed. Twenty-one horses had several problems contributing to pain and poor performance.

Horse Number	Source(s) of Pain Reduced by Diagnostic Anaesthesia	Residual Clinical Signs and Suspected Cause of Persistent Pain	RHpE Score before Diagnostic Anaesthesia	RHpE Score after Diagnostic Anaesthesia
13	Bilateral hindlimb PSD, LSI joint region pain	Reduced range of motion of thoracolumbosacral region. Severe impinging spinous processes T13-L3, osteoarthritis APJs T17-L1	8	3
26	Bilateral hindlimb PSD, LSI joint region pain	Reduced range of motion of neck and persistent but reduced head tilt. Dorsal subluxation of head of T1	11	4
37	LSI joint region pain. Degenerate lumbosacral disk	Uncomfortable in sitting trot, broke in canter. Failure to remove all pain	9	4
39	Bilateral hindlimb PSD, LSI joint region pain	Stumbled right forelimb, neck stiffness, head and neck tilt when ridden on long rein. Cervical radiculopathy	10	2
42	Bilateral front foot pain, bilateral hindlimb PSD, LSI joint region pain	Crooked canter on forehand and head tilt and neck stiffness. Psoas major and braciocephalicus muscle pain, osteoarthritis of Ce6/7 and Ce7/T1 APJs	9	3
43	Bilateral hindlimb PSD, LSI joint region pain	Reluctant to canter, head and neck tilt in canter, neck pain. Subluxation Ce6/7 and osteoarthritis of Ce6/7 APJs	9	8
50	Bilateral hindlimb PSD, LSI joint region pain	Short stepping forelimb gait, resistant to bend neck. Severe osteoarthritis of Ce6/7 and Ce7/T1 APJs	9	6
56	Bilateral hindlimb PSD, LSI joint region pain	Very stiff neck and short stepping forelimb gait, canter > trot, broke in canter, much better on long rein, pain on pressure over Ce6/7 APJs. Severe osteoarthritis of Ce6/7 APJs and narrowed IVF	9	3
67	Bilateral hindlimb PSD	Left forelimb lameness and severe head tilt (canter > trot). Subluxation Ce7/T1 and severe osteoarthritis of Ce6/7 and Ce7/T1 APJs	10	6
73	Right stifle pain, left front PSD, right front PSD and proximal lesion of DDFT; osteoarthritis antebrachiocarpal joints (LF and RF)	Still short stepping but hugely improved compared with baseline; likely myofascial restrictions	10	4
75	Bilateral hindlimb PSD	Left forelimb lameness and stumbling provoking head tilt, associated with neurogenic atrophy of left pectoral muscles	7	5
76	Not applicable	Left forelimb lameness and severe stumbling, which provokes head and neck tilt. Subluxation Ce6/7 and discospondylosis	9	8
77	Not applicable	Idiopathic hopping-type forelimb lameness. Osteoarthritis Ce6/7 and Ce7/T1 APJs	6	6
87	Bilateral hindlimb PSD, LSI joint region pain	Right forelimb lameness and head tilt when ridden to a contact, stiff neck, short forelimb steps in canter. Cervical radiculopathy	11	12
108	Bilateral hindlimb PSD, LSI joint region pain	Shortened forelimb step length, canter >> trot, pain on pressure over Ce6/7 APJs, left > right. Severe osteoarthritis Ce6/7 APJs	8	2
116	Bilateral HL PSD; medial branch SL injury RH	Lacked hindlimb impulsion in canter, reactive to pressure over tubera sacrale and lumbar epaxial muscle hypertension; mildly ataxic after SI blocks, therefore not reassessed	13	3
126	Bilateral HL PSD	Canter left worse than canter right; mildly ataxic after SI blocks, therefore not reassessed when ridden	8	4
131	Bilateral hindlimb PSD, LSI joint region pain	Restricted gait (although greatly improved compared with baseline); cause of residual discomfort could not be determined	12	5
134	LSI joint region pain	Left forelimb lameness when ridden to a contact, head tilt, pain on pressure over left Ce6/7 APJs. Cervical radiculopathy	9	9
136	Bilateral HL PSD	Canter still lacked hindlimb impulsion, but owner thrilled by improvement and did not wish to proceed further	11	1
137	Bilateral hindlimb PSD, LSI joint region pain	Canter still lacked hindlimb impulsion, but owner considered horse hugely better and did not wish to proceed further	5	1
142	Bilateral hindlimb PSD	Still broke spontaneously from canter to trot; horse noncompliant, so SI joint blocks not performed	13	4
145	Bilateral hindlimb PSD, LSI joint region pain; left front foot pain	Idiopathic hopping type right forelimb lameness: only lame when ridden to a contact. Cervical radiculopathy	7	3

PSD, proximal suspensory desmopathy; LSI, lumbosacroiliac; DDFT, deep digital flexor tendon; Ce 6, 6th cervical vertebra; Ce7, 7th cervical vertebra; T1, 1st thoracic vertebra; L3, 3rd lumbar vertebra; APJ, articular process joint; IVF, intervertebral foramen; LF, left forelimb; RF, right forelimb; SI, sacroiliac.

### 3.4. Other Observations

Teeth grinding was observed in 13 (8.7%) horses before the interventions. Thirty-one (20.7%) horses made an abnormal respiratory noise, usually while cantering. There was a delayed recovery of the resting respiratory rate after exercise relative to the work intensity, environmental temperature and fitness in six (4.0%) horses and sweating disproportionate to the work intensity, environmental temperature and fitness in nine (6.0%) horses. Thirteen (9%) horses displayed an abnormal posture after exercise. The frequency of each of these observations reduced substantially after the interventions. Teeth grinding was heard in only one (0.7%) horse, and an abnormal respiratory noise was heard in eight (5.3%) horses. There was a delayed recovery of the respiratory rate after exercise in one (0.7%) horse and sweating disproportionate to the work intensity, environmental temperature and fitness in one (0.7%) horse. No horse had an abnormal posture after exercise. Twenty-seven (18.0%) horses snorted several times soon after resuming work after the interventions resulted in substantial improvements in lameness and the quality of the gaits.

The riders frequently volunteered comments about what they felt during ridden exercise after the interventions; for example, a more symmetrical rein tension, increased range of motion of the thoracolumbosacral region, reduced rotation of their pelvis during canter and an increased responsiveness to cues.

## 4. Discussion

This is the first large-scale study which has systematically documented the performance of ridden horses after diagnostic anaesthesia. In most horses, lameness was abolished and there was improvement in the quality of canter, that was associated with substantial reductions in RHpE scores, which was in accordance with our hypothesis. This verifies previous observations that there is a causal relationship between pain and the behaviours of the RHpE [16,17] and that the majority of behaviours are not habitual.

### 4.1. Lameness Grading

As anticipated, there was no association between the maximum lameness grade assigned during ridden exercise and the RHpE score. The maximum lameness grade during ridden exercise was unlikely to reflect the degree of discomfort experienced by a horse, because 147/150 (98%) horses were lame in more than one limb and, in such circumstances, grading cannot reflect the lameness severity [27]. Moreover, horses adapt to lameness by reducing the range of motion of the thoracolumbosacral region, reducing step length and suspension and increasing duty factor to try to minimise discomfort and effectively conceal lameness [30,31,32,33,34,35,36,37,38]. A lameness grade could not be assigned to those horses with bilaterally symmetrical gaits under all circumstances, although the direction of saddle slip (a saddle most frequently slips to the side of the lame(r) hindlimb) [28] or the ease with which a horse performed movements on the left versus the right reins provided indicators to which limb might be the most uncomfortable.

It was notable that in some horses, the quality of the canter deteriorated despite the resolution of lameness. This reflects a change in the primary source of pain and its influence on the canter which, in contrast to a trot, is an asymmetrical gait that is initiated by the trailing hindlimb bearing weight alone. This observation also highlights the need to evaluate the movement of horses in all paces before and after diagnostic anaesthesia. The residual abnormalities of canter are usually resolved after infiltration of mepivacaine around the sacroiliac joints, as has previously been documented [2]. This local anaesthetic technique is not specific and there is also potential to influence, but not necessarily abolish, pain associated with the lumbosacral joints and the cranial gluteal, obturator and sciatic nerves and the lumbosacral plexus [39,40].

### 4.2. Rider Factors

In a previous study, it was demonstrated that rider skill may influence the gait quality, the presence or absence of lameness and ridden horse behaviour [41]. For some horses, there was a change in the manifestation of specific behaviours of the RHpE, but the median RHpE scores did not change with two different riders. In the current study, the relative skills of the usual rider (ranging from inexperienced amateur riders to highly skilled professionals) and a technician varied. Some riders, knowingly or unknowingly, had a potentially adverse influence on the horse through a lack of balance, coordination, fitness, straightness, an inability to keep their hands still, over-restrictive hand cues or failure to apply appropriate leg cues. When a horse was ridden by the usual rider throughout the investigation, it was sometimes necessary to advise the rider to ride forwards more, keep their hands still by positioning the fifth digit of each hand on the dorsal part of the horse’s neck or to hold a neck strap, or to ride in a ‘two-point’ position to improve their synchrony of movement with the horse.

If a technician rode a horse throughout the diagnostic procedures, the usual rider (if present) was encouraged to ride the horse at the end so that they could appreciate the difference in the horse’s quality of movement and responsiveness to the rider’s cues. The riders (both the usual rider and a technician) were often astonished by the changes in the horse’s movement patterns and rideability after the successful resolution of pain. The usual rider frequently observed that their horse had never previously moved so well throughout their current ownership, which indicates the likely chronicity of an underlying problem. The improved education of riders and trainers about how to differentiate between genuine training problems versus problems arising secondary to discomfort might facilitate an earlier diagnosis and prevention of secondary musculoskeletal adaptations.

The resolution of a horse’s pain allowed riders (the technicians or the usual rider) to unconsciously improve their posture on the horse. The riders usually commented that the horses were easier to ride and were more responsive to leg and rein cues. There were also comments such as an absence of a jarring impulse being generated through the rider’s vertebral column, reflecting increased range of motion of the horse’s thoracolumbosacral region, or the absence of shoulder discomfort because a horse was taking a more even rein contact. This highlights the importance of riders and trainers recognising that pain-free horses are both more rideable and enable riders to be in a better position and to experience less discomfort.

### 4.3. Ridden Horse Pain Ethogram Assessments before Interventions

As in previous studies [13,14,16,17,42], a minority of lame horses in the current study had RHpE scores <8/24; however, the majority had a score of ≥8/24, which provides further evidence that a score of ≥8/24 is highly likely to reflect musculoskeletal pain. The most frequently displayed behaviours were an intense stare for ≥5 s, repeated head tilt, repeated movement of the head up and down, repeated hindlimb toe drag or forelimb or hindlimb stumbling and ears back for ≥5 s. However, all 24 behaviours were observed, which reflects the different ways in which horses react to pain.

### 4.4. Changes in Behaviour after Diagnostic Anaesthesia and Removal of Pain

Each horse acted as its own control (a repeated measures study design), with the only variables being diagnostic anaesthesia ± change of saddle. There was a reduction in the frequency of occurrence of all 24 behaviours of the RHpE after the interventions. The reduction in frequency for head behind vertical >10° for ≥10 s was only 15.3%, which is in accordance with previous observations [16,17]. This may reflect that in some horses there is a change from head above vertical >30° for ≥10 s to head behind vertical >10° for ≥10 s after removal of underlying pain, as observed in 13 of 62 (21%) horses in the current study. It may also reflect a current trend to train horses with the head behind a vertical position [43,44,45,46]. As previously documented [16,17] a crooked tail persisted in 19% of horses which may be the result of abnormal myofascial tension [20]. Hindlimb toe drag or stumbling was reduced in frequency by approximately 34% to 30%. It is well recognised that perineural anaesthesia in hindlimbs may induce a hindlimb toe drag [47], which reflects proprioceptive dysfunction, so this result was not unexpected.

### 4.5. Residual Lameness or Discomfort

Subtle hindlimb asymmetry (<grade 1/8 [27]) was observed in a small proportion of the Warmblood dressage horses after substantial improvement in the baseline lameness and improvement in the RHpE scores. This residual asymmetry may represent inherent laterality [48] or failure to completely remove the pain causing lameness. Persistent abnormal myofascial tension may also have been a contributory factor [49,50].

More obvious residual gait abnormalities were observed in 23 horses (15%) (Table 4), the majority of which were considered to be related to cervical radiculopathy [51,52,53] and/or neck pain associated with subluxation of caudal cervical vertebrae and/or severe osteoarthritis of the caudal cervical or cranial thoracic articular process joints. The degree of change in the RHpE scores before and after the removal of other sources of pain was variable (Table 4), which reflects the relative contributions of each source of pain to the overall clinical presentation.

### 4.6. Other Observations during Riding Exercise

Teeth grinding, an abnormal respiratory noise during ridden exercise, delayed recovery of the resting respiratory rate, excessive sweating and an abnormal posture after ridden exercise were not features that were included in the original development of the RHpE but are considered likely to reflect discomfort in some horses. This is substantiated by the reduction in these observations after the interventions, with each horse acting as its own control (repeated measures) for these subjective assessments. These differences were not analysed statistically because of the small numbers and low statistical power.

Transient snorting was occasionally observed during the original assessment but was heard in 18% of horses after a reduction in pain. We previously observed this [16] but did not quantify the observation. This is likely to reflect improved comfort and a positive emotional state [54,55].

### 4.7. Saddle Fit

There was a high frequency of occurrence (37%) of ill-fitting saddles, which influenced performance despite a large proportion of horses having undergone professional saddle fit checks within the previous one to three months. However, many riders were unaware of the professional qualifications of the saddle fitter (for example, Society of Master Saddlers Qualified Saddle Fitter). In all horses, an improvement in behaviour and/or gait was observed after changing their current saddle to a better fitting saddle, although paradoxically, in some horses, lameness became apparent (Table 1) or deteriorated, which was associated with a subjectively assessed increased range of motion of the thoracolumbosacral region and increased oscillation of the tail.

In the current study, there was no association between an ill-fitting saddle and the total RHpE score. This result contrasts with a previous study of horses which were in full work and believed by their owners to be working comfortably, although 62% were lame when ridden [4]. The horses with a saddle with tight tree points had significantly higher RHpE scores compared with the horses with a well-fitting saddle. The difference in results probably reflects the difference between the studies in the relative contribution to pain by the saddle or primary musculoskeletal injury, with horses in this study having a greater component of pain arising from the latter.

Saddle fit was not ideal for the horse in a larger proportion of the cohort, manifest as excessive bouncing or oscillation during ridden exercise, but this was considered unlikely to have a major influence on the performance of these horses. A high frequency of occurrence of ill-fitting saddles was previously documented in both the United Kingdom [22] and Switzerland [56]. Moreover, for some riders, the saddle was not large enough, which resulted in the rider’s weight being concentrated over the caudal third of the saddle. An association between a rider sitting on the caudal third of the saddle and RHpE scores was previously documented [4]. Saddle fit for both the horse and the rider was addressed in the subsequent management programme for each horse–rider combination. When necessary, advice was also given about rider posture, coordination, and fitness.

### 4.8. Limitations of the Study

As with many clinical investigations, this study has limitations. All the assessments were subjective. The use of objective gait analysis has some limitations in horses with lameness in several limbs, especially when gait abnormalities are only seen when a horse is performing specific movements when ridden, such as shoulder in. A bilaterally symmetrical shortened hindlimb step length with a lack of hindlimb impulsion and engagement may be interpreted as normal when using inertial measurement units, whereas substantial improvement in gait quality and ridden horse behaviour can be observed after the successful resolution of hindlimb pain by diagnostic anaesthesia [57]. Variations in canter have not been investigated objectively to date.

A single trained observer performed all the evaluations; this resulted in consistency in the assessment techniques but resulted in the potential for consistent bias. However, there was an independent assessment of the rideability of the horses and their gait quality from all the riders. It is, however, acknowledged that the riders could also be biased and may have been influenced by the observations of the principal assessor. We have previously shown good agreement among veterinarians applying the RHpE in real time and also between the real-time application of the RHpE and a retrospective analysis of video recordings [58].

There was not a standardised exercise test for all horses because it was previously demonstrated that a horse needs to be seen performing the full repertoire of movements that it is expected to perform based on its work discipline and work level for the RHpE to be used accurately. However, each horse effectively acted as its own control (a repeated measures study).

There were too few horses with only one diagnosis to investigate if there was any relationship between a particular source of pain and the RHpE scores. Gastroscopy was not performed to determine the presence of squamous or glandular stomach ulcers. However, many owners reported that their horses had previously undergone gastroscopy and treatment with omeprazole (up to five courses) and management changes, with little change in ridden horse performance. Nonetheless, the contribution of visceral pain to ridden horse behaviour cannot be excluded [59].

The results of lameness assessment in hand and on the lunge are not presented. It is beyond the scope of the current study to make comparisons between the gait abnormalities observed when ridden compared with the gait abnormalities observed when the horses were not ridden. Nonetheless, it was clear during the clinical assessments that the riders’ problems could only be fully understood by observing the horses when ridden, many of which did not show overt lameness in hand. All the available horses were included in the study, so this was not a closed cohort. It is important to recognise that ultimately the aim was to reach as accurate a diagnosis as possible, so that future treatment and management could be optimised, to give each horse the maximum chance for return to full athletic function. 

## 5. Conclusions

This study highlights the importance of including ridden exercise in investigations of low-grade lameness and poor performance. Horses should be evaluated at all gaits and when performing all movements in their current work repertoire. In most horses, the RHpE score was ≥8/24 before diagnostic anaesthesia, despite lameness in the conventional sense being low-grade or absent, emphasising the value of the RHpE for recognition of the presence of musculoskeletal pain. The substantial reduction in RHpE scores after diagnostic anaesthesia verifies the value of the RHpE as a tool to determine if most of the pain causing compromises in performance has been resolved. The high prevalence of ill-fitting saddles, which adversely affected performance in these horses, emphasises the importance of educating riders and trainers about the importance of correct saddle fit for both the horse and rider for optimal performance.

## Figures and Tables

**Table 1:** Summary of the 24 behaviours of the Ridden Horse Pain Ethogram (adapted from Dyson et al. [13]).

1. Repeated changes in head position up and down, not in rhythm with the trot
2. Head tilted repeatedly
3. Head in front of vertical (>30°) for ≥10 s
4. Head behind vertical (>10°) for ≥10 s
5. Repeated changes in head position from side to side
6. Ears rotated back behind vertical (both or one only) for ≥5 s; repeatedly lay flat
7. Eye lids closed or half closed for 2–5 s
8. Sclera exposed repeatedly
9. Intense stare (glazed expression, ‘zoned out’) for ≥5 s
10. Mouth opening ± shutting repeatedly with separation of teeth for ≥10 s
11. Tongue exposed, protruding or hanging out and/or moving in and out repeatedly
12. Bit pulled through the mouth on one side (left or right) repeatedly
13. Tail clamped tightly to middle or held to one side
14. Tail swishing: large movements, repeatedly up and down/side to side/circular; repeatedly during transitions
15. A rushed gait (frequency of trot steps >40/15 s); irregular rhythm in trot or canter; repeated changes of speed in trot or canter
16. Gait too slow (frequency of trot steps <35/15 s); passage-like trot
17. Hindlimbs do not follow tracks of forelimbs but repeatedly deviated to left or right; on 3 tracks during trot or canter
18. Canter repeated incorrect strike off with wrong forelimb leading; repeated change in leg in front and/or behind (disunited or cross-cantering)
19. Spontaneous changes in gait (e.g., breaks from canter to trot or trot to canter)
20. Stumbles or trips more than once in front or behind; repeated bilateral hindlimb toe drag
21. Sudden change in direction against rider’s cues; spooking
22. Reluctance to move forwards (has to be kicked ± verbal encouragement); stops spontaneously
23. Rearing (both forelimbs off the ground)
24. Bucking or kicking backwards (one or both hindlimbs)

**Table 2:** An example of a summary of clinical observations and interventions performed for a horse investigated for poor performance during ridden exercise.

Order of Observations and Interventions	Clinical Observation	Intervention
1	Tight tree points of saddle; concavities in epaxial muscles under cranial saddle region; lack of sweating under cranial third of each saddle panel No lameness observed in hand or on the lunge, but short stepping hindlimb gait on the lunge Head movement up and down during ridden exercise Lack of hindlimb impulsion and engagement in trot and canter; reduced range of motion of the thoracolumbosacral region Ridden Horse Pain Ethogram (RHpE) score 9/24	Change in saddle
2	Lowered head and neck and position of head and neck more stable Head behind vertical Increased range of motion of the thoracolumbosacral region Lame left hind grade 3/8; no localising clinical signs Uniform sweat pattern under saddle	Perineural anaesthesia of left hind plantar (at junction of proximal 2/3 and distal 1/3 of metatarsal region) and plantar metatarsal nerves (distal to distal aspect of second and fourth metatarsal bones)
3	Lame left hind grade 4/8	Perineural anaesthesia of left hind deep branch of the lateral plantar nerve
4	Lame right hind grade 3/8	Perineural anaesthesia of right hind deep branch of the lateral plantar nerve
5	Nonlame but quality of canter deteriorated; more ‘on the forehand’; rider’s pelvis rotated in right lead canter	Infiltration of mepivacaine (2 × 8 mL) around left and right sacroiliac joints
6	Nonlame; canter quality hugely improved; rider’s pelvis no longer rotated RHpE score 1/24	

**Table 3:** Distribution in lameness groups of 150 horses assessed when ridden as part of a poor performance assessment.

Lameness Group	Number	Percentage
Bilateral hindlimb lameness and lumbosacroiliac joint region pain	56	37.3
Bilateral hindlimb lameness and lumbosacroiliac joint region pain and bilateral/unilateral forelimb lameness	25	16.7
Bilateral hindlimb lameness	15	10.0
Lumbosacroiliac joint region pain	15	10.0
Bilateral hindlimb lameness and lumbosacroiliac joint region pain and other (e.g., impinging spinous processes)	11	7.3
* Other (<5 per group)	13	8.7
Unilateral or bilateral forelimb lameness	8	5.3
Bilateral hindlimb lameness and unilateral forelimb lameness	7	4.7

* Other = bilateral hindlimb (HL) lameness and other (e.g., cervical pain), *n* = 4 (3%); lumbosacroiliac (LSI) joint region pain and unilateral or bilateral forelimb (FL) lameness, *n* = 2 (1%); LSI joint region pain and other (e.g., impinging spinous processes), *n* = 3 (2%); bilateral HL lameness and bilateral FL lameness, *n* = 1 (0.7%); impinging spinous processes, *n* = 1 (0.7%); bilateral FL lameness and unilateral HL lameness, *n* = 1 (0.7%); ill-fitting saddle for horse and rider (not lame after change in saddle), *n* = 1 (0.7%).

## Data Availability

Anonymised data are available from the authors upon reasonable request.

## Supplementary Materials

The following supporting information can be downloaded at https://www.mdpi.com/article/10.3390/ani13121940/s1: Table S1: Changes in frequency of occurrence of each of the 24 behaviours of the Ridden Horse Pain Ethogram (RHpE) in 150 horses undergoing investigation of poor performance before and after diagnostic anaesthesia ± saddle change. N = number, % = percentage.
