# Supplementary material for: Application of the Ridden Horse Pain Ethogram to 150 Horses with Musculoskeletal Pain before and after Diagnostic Anaesthesia
Source: Animals (Basel). 2023 Jun 9;13(12):1940. doi: 10.3390/ani13121940 (PMC10295347; doi:10.3390/ani13121940)
Supplement: Supplementary file 1 [file animals-13-01940-s001.zip › animals-2432288-supplementary.pdf]

Supplementary information

Table S1 Changes in frequency of occurrence of each of the 24 behaviours of the Ridden Horse Pain Ethogram (RHpE) in 150 horses undergoing investigation of poor performance before and after diagnostic anaesthesia ± saddle change. N=number, %= percentage.

**RHpE behaviour**	**Before (N)**	**After (N)**	**Before (%)**	**After (%)**	**Difference (before - after %)**
Repeated changes of head position	103	17	69	11	57.3
Head tilted or tilting repeatedly	105	24	70	16	54.0
Head in front of vertical	63	11	42	7	34.7
Head behind vertical	74	51	49	34	15.3
Head position changes regularly	54	6	36	4	32.0
Ears rotated back behind vertical or flat	91	15	61	10	50.7
Eye lids closed or half closed	31	2	21	1	19.3
Sclera (white of eye) exposed	40	2	27	1	25.3
Intense stare	128	37	85	25	60.7
Mouth opening	77	16	51	11	40.7
Tongue exposed	43	5	29	3	25.3
Bit pulled through the mouth on one side	42	6	28	4	24.0
Tail clamped tightly to middle or held to one side	51	29	34	19	14.7
Tail swishing large movements	77	23	51	15	36.0
A rushed gait	28	1	19	1	18.0
Gait too slow	6	0	4	0	4.0
Hindlimbs do not follow tracks of forelimbs	86	21	57	14	43.3
Canter repeated leg changes	21	1	14	1	13.3
Spontaneous changes of gait	71	11	47	7	40.0
Stumbles or trips/catches toe repeatedly	96	45	64	30	34.0
Sudden change of direction; spooking	22	2	15	1	13.3
Reluctant to move forward, stops spontaneously	64	10	43	7	36.0
Rearing	6	0	4	0	4.0
Bucking or kicking backwards	18	0	12	0	12.0
